# Immune cells, inflammatory proteins, and sepsis: A mediation Mendelian randomization study

**DOI:** 10.1097/MD.0000000000043779

**Published:** 2025-08-08

**Authors:** Yan Zhang, Weiwei Xu, Wenqi Huang, An Zhang

**Affiliations:** a Department of Critical Care Medicine, The Second Affiliated Hospital of Chongqing Medical University, Chongqing, China; b Department of Endocrine and Metabolic Diseases, The Second Affiliated Hospital of Chongqing Medical University, Chongqing, China.

**Keywords:** immune cell traits, inflammatory proteins, linkage disequilibrium score, Mendelian randomization, sepsis

## Abstract

Immune cells are known to be associated with sepsis. However, whether these associations represent a causal relationship and whether inflammatory proteins act as mediators remain unclear. A mediation Mendelian Randomization (MR) approach was employed to assess the correlation between immune cells and sepsis, along with the mediating effects of inflammatory proteins in this relationship. Inverse variance weighting (IVW) was used as the main statistical method, with MR-Egger and weighted median serving as supplements in the preliminary MR. Sensitivity analyses were implemented using Cochrane *Q* test, MR-Egger intercept, Mendelian Randomization Pleiotropy RESidual Sum and Outlier and leave-one-out analysis. Subsequently, we performed replication MR, meta-analysis, Reverse MR, and linkage disequilibrium score (LDSC) regression to thoroughly verify causation. In addition, we explored whether inflammatory proteins act as mediating factors in the pathway from the immune cells to sepsis. After conducting a meta-analysis of the discovery and replication cohorts, there were 4 risk and 7 protective causal effects between genetic liability in immune cells and sepsis, with no evidence of reverse causality. Among the 92 inflammatory proteins investigated, only 2 were found to be associated with sepsis. However, inflammatory proteins did not act as mediating factors. This study elucidated the critical role of specific immune cell traits in the progression of sepsis. Our findings provide a foundation for future research into targeted immunomodulatory therapies, potentially improving patient outcomes in sepsis, and offering new insights into the complex immunological dynamics of this condition.

## 1. Introduction

Sepsis represents a critical global health challenge and is characterized by potentially fatal organ dysfunction resulting from a dysregulated host inflammatory and immune response to infection. This conceptual shift from focusing solely on the causative pathogen to emphasizing the host immune response has been pivotal in advancing our understanding of sepsis. In 2017, an estimated 49 million cases of sepsis were reported worldwide, leading to approximately 11 million deaths, accounting for 20% of global mortality that year.^[1]^ Epidemiological data further highlight the severity of the condition, with intensive care unit (ICU) and hospital mortality rates for sepsis patients reaching 26% and 35%, respectively.^[2]^ Beyond its profound impact on patient health, sepsis imposes a substantial burden on healthcare systems and the economy.

The pathogenesis of sepsis is complex, and involves intricate interactions between various immune cells and inflammatory mediators. Key immune cells, including monocytes/macrophages, neutrophils, and lymphocytes, play a crucial role in the initiation and progression of sepsis. These cells are not only essential for pathogen recognition and clearance but also regulate inflammatory responses through cytokine release. For instance, reduced monocyte human leukocyte antigen-DR (HLA-DR) expression in sepsis patients has been associated with poor prognosis, whereas delayed neutrophil apoptosis has been linked to exacerbated inflammatory responses and tissue damage.^[3–6]^ Glucocorticoid administration has shown efficacy in mitigating the hyperinflammatory response in sepsis, thereby improving the clinical outcomes.^[7,8]^ Moreover, numerous studies^[9–13]^ have documented elevated levels of inflammatory factors in patients with sepsis, which are closely correlated with disease severity and prognosis. These inflammatory mediators, which are produced by immune cells, create a complex interaction network that further influences immune cell function and activity.^[14]^ Targeted therapeutic strategies, such as anti-IL-1β treatment, have shown promise for improving sepsis outcomes.^[15,16]^ Inflammatory proteins, including tumor necrosis factor (TNF-α), IL-6, and IL-1β, are integral to the inflammatory response in sepsis, driving immune cell activation and migration and modulating vascular permeability and coagulation.^[17–20]^

However, it is important to recognize that these observational studies are inherently limited in their ability to infer causal relationships. Consequently, the causal link between immune cells and sepsis as well as the potential mediating role of inflammatory proteins remains uncertain. This underscores the need for a more in-depth exploration of the causal relationships within this context.

Despite substantial progress in elucidating the roles of immune cells and inflammatory proteins in sepsis, several critical questions remain unresolved. First, while immune cells are known to play a pivotal role in the pathogenesis of sepsis, the precise mechanisms by which they influence an individual’s susceptibility to sepsis are not fully understood. Second, inflammatory proteins are key players in the immune response during sepsis; however, whether they act as primary mediators of the impact of immune cells on sepsis risk remains to be determined. Third, although immunotherapy has shown promise for other inflammatory and infectious diseases, its efficacy and feasibility in sepsis management are still under investigation. Addressing these questions is essential to bridge the gap in our understanding of the causal mechanisms underlying sepsis and fully realize the potential of immunotherapy in mitigating its impact.

To address these questions, we conducted a comprehensive Mendelian randomization (MR) analysis to investigate the causal relationships between immune cells, inflammatory proteins, and sepsis, including its subtypes (such as those 28-day death, under 75, 28-day death in Critical Care and critical care, streptococcal septicemia, and puerperal sepsis). We utilized linkage disequilibrium (LD) score regression (LDSC) to assess the genetic correlation between immune cells and susceptibility to sepsis. Subsequently, mediation MR analysis was performed using a panel of 92 inflammatory proteins to elucidate the potential pathways through which immune cells may influence sepsis risk. This approach aims to enhance our understanding of the immunological mechanisms underlying sepsis and identify new therapeutic targets for sepsis immunotherapy in the future.

## 2. Materials and methods

### 2.1. Study design

**Figure 1.**
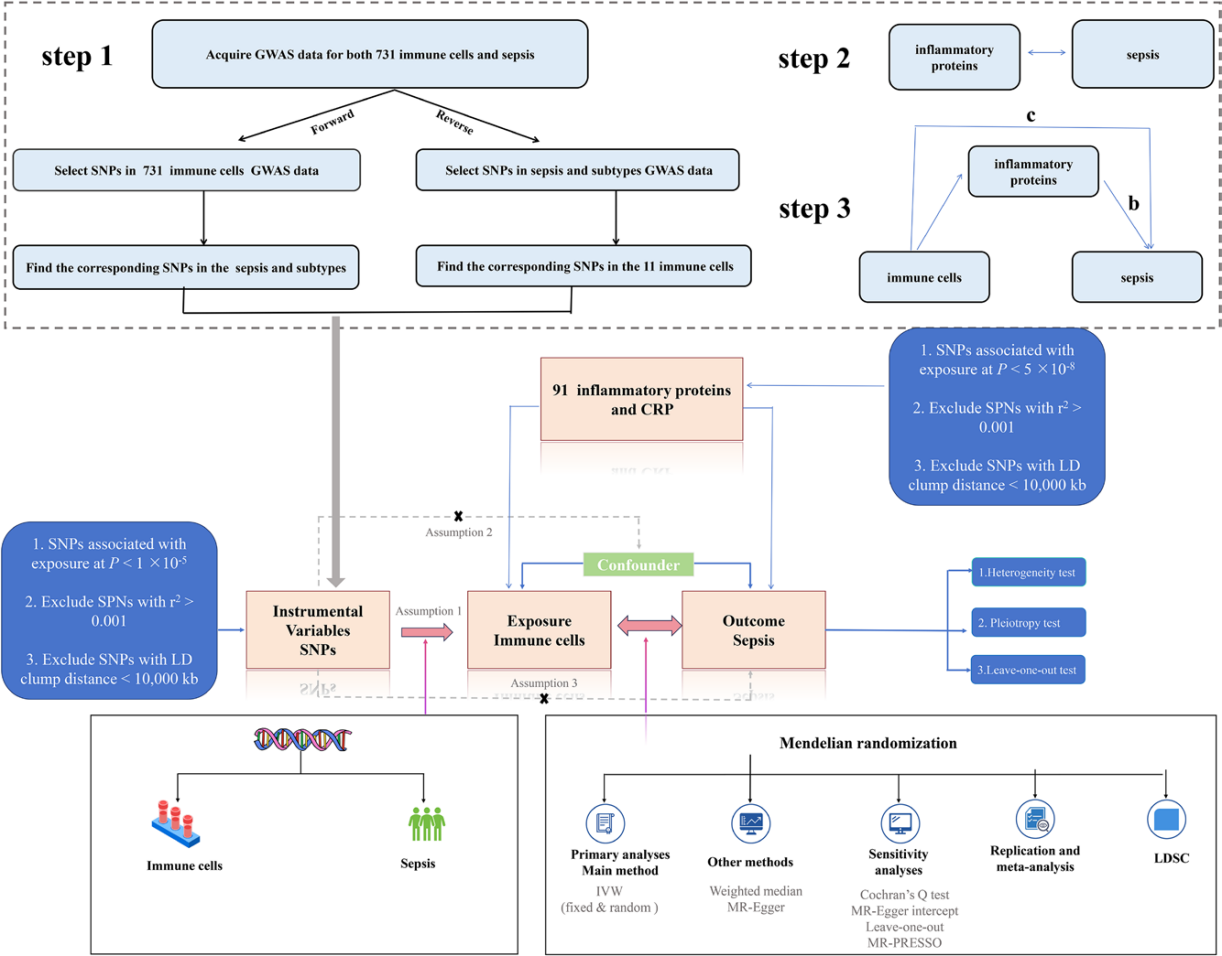
Overview and assumptions of the Mendelian randomization study design.

This study was structured into 3 primary components, as illustrated in Figure 1: analysis of the causal effects of 731 immune cell traits on sepsis and its subtypes (step 1), analysis of the causal effects of 91 inflammatory proteins and C-reactive protein (CRP) on sepsis (step 2), and mediation analysis of inflammatory proteins in the pathway linking immune cells to sepsis (step 3). In our approach, single-nucleotide polymorphisms (SNPs) were used as instrumental variables (IVs). MR is based on 3 core assumptions^[21]^: genetic variation is directly associated with the exposure of interest; genetic variation is independent of any confounders between the exposure and the outcome; and genetic variation influences the outcome solely through exposure, with no alternative pathways involved.^[22]^ This study adhered to the Strengthening the Reporting of Observational Studies in Epidemiology using Mendelian Randomization (STROBE-MR) guidelines, as detailed in the checklist provided in the Supplementary Material.^[23]^

### 2.2. Immunity-wide genome-wide association study (GWAS) data sources

The genome-wide association study (GWAS) summary statistics for each immune trait utilized in this study are publicly available from the GWAS Catalog, with accession numbers ranging from GCST0001391 to GCST0002121.^[24]^ In total, 731 immunophenotypes were included, including absolute cell (AC) counts (n = 118), median fluorescence intensities (MFI), which reflect surface antigen levels (n = 389), morphological parameters (MP) (n = 32), and relative cell counts (n = 192). Specifically, median fluorescence intensities, absolute cell, and relative cell traits included features related to B cells, conventional dendritic cells, various maturation stages of T cells, monocytes, myeloid cells, TBNK (T cells, B cells, natural killer cells), and Treg panels. Morphological parameters traits encompassed the CDC and TBNK panels. The original GWAS on immune traits was conducted using data from 3757 Sardinian individuals, with no overlapping cohorts. Approximately 22 million SNPs were genotyped using high-density arrays and subsequently imputed with a Sardinian sequence-based reference panel.^[25]^ Associations were tested after adjusting for relevant covariates, including sex, age, and the square of age (age^2^), as shown in Table S1 (Supplemental Digital Content, https://links.lww.com/MD/P612).

### 2.3. Inflammatory protein GWAS data sources

The data for inflammatory proteins were obtained from a study by Zhao et al (Table S1, Supplemental Digital Content, https://links.lww.com/MD/P612).^[26]^ This study conducted a comprehensive assessment of the genetic effects on inflammation-related proteins through a genome-wide study of protein quantitative trait loci (pQTL). The study included 14,824 participants, and 91 plasma proteins were measured using the Olink panel. Additionally, C-reactive protein (CRP), a commonly studied inflammatory marker, was not included in the study by Zhao et al.^[26]^ We used summary genetic information of European ancestry for CRP-related genetic variants provided by Said et al, which included 575,531 individuals of European ancestry (Table S1, Supplemental Digital Content, https://links.lww.com/MD/P612).^[27]^

### 2.4. GWAS data sources for sepsis and sub-phenotypes of sepsis

Summary statistics for sepsis and its sub-phenotypes were generated using summary-level data obtained from the UK Biobank (UKBB) (https://biobank.ctsu.ox.ac.uk) and the FinnGen consortium (https://www.finngen.fi/en/access_results) (Table S2, Supplemental Digital Content, https://links.lww.com/MD/P612). Data from the UK Biobank were used as the discovery cohort, while data from FinnGen were used as the replication cohort.

The UK Biobank study is a large prospective cohort study that recruited more than 500,000 individuals aged > 40 years between 2006 and 2010.^[28]^ This dataset encompassed sepsis and its subgroups (including those 28-day death, under 75, 28-day death in Critical Care and critical care). The dataset consisted of 11,643, 1896, 11,568, 347, and 1380 sepsis cases, along with 474,841, 484,588, 451,301, 431,018, and 429,985 controls, respectively. The discovery cohort cases were included if the code appeared in the primary or secondary diagnostic category in the hospital episode statistics data provided by the UK Biobank. This analysis was performed using Regenie v2.2.4, adjusting for age, sex, genotyping chip, and the initial 10 principal components.^[29]^ Sepsis admissions were identified in the UK Biobank’s ICD code-linked secondary care data using ICD-10 codes A02, A39, A40, and A41, consistent with recent literature.^[30,31]^ All the participants in these studies were of European ancestry. Detailed information is provided in Table S2 (Supplemental Digital Content, https://links.lww.com/MD/P612).

The FinnGen consortium is a large public-private partnership aimed at collecting and analyzing genomic and health data from 500,000 Finnish biobank participants. We used the R10 data release of FinnGen, and genome-wide association analyses for each trait were adjusted for sex, age, genotyping batch, and the first ten genetic principal components. This dataset included sepsis (13,531 cases, 363,227 controls), (streptococcal septicemia (2543 cases, 363,227 controls) and puerperal sepsis (4342 cases, 220,773 control), which served as the replication cohort.

### 2.5. Selection of IVs

Consistent with recent research,^[24,32,33]^ the significance threshold for selecting IVs for each immune cell trait was set at 1 × 10^−^5, as the appropriate IVs could not be reliably identified with a more stringent threshold of *P* < 5 × 10^−^8. For inflammatory proteins, a more stringent significance level of 5 × 10^−8^ was applied.

Data from the European samples of the 1000 Genomes Project were utilized as the reference panel to calculate the LD between SNPs. SNPs with *r*^2^ < 0.001 were retained using a clumping window of 10,000 kb. To ensure consistency, the exposure and outcome datasets were harmonized and palindromic SNPs with intermediate allele frequencies were excluded.

The *F*-statistic was calculated to assess the strength of the genetic instruments, with SNPs exhibiting an *F*-statistic <10 excluded due to inadequate instrument strength. The *F*-statistic was calculated using the formula (β/SE)2, where β represents the estimated genetic effect of each SNP and SE denotes the standard error of β.

### 2.6. Discovery MR analysis

To estimate the causal effects of immune cells and inflammatory proteins on sepsis, we conducted 2-sample MR analyses for each, corresponding to Steps 1 and 2 in Figure 1. We primarily utilized the inverse variance weighted (IVW) method, which combines the Wald ratios of individual SNPs with the outcome to produce a pooled causal estimate. In addition to IVW, we employed supplementary MR analyses, including the MR-Egger regression and the weighted median method, to provide more robust estimates under a broader range of scenarios. The MR-Egger regression offers a test for unbalanced pleiotropy and accounts for heterogeneity, although it requires a larger sample size when underexposure variation is present.^[34]^ The weighted median method, on the other hand, yields consistent effect estimates provided that at least half of the weighted variance is derived from valid instruments unaffected by horizontal pleiotropy.^[35]^ Heterogeneity was assessed using Cochrane *Q* test,^[36]^ where *P* < .05 indicated the presence of heterogeneity among the SNPs. Results with an MR-Egger intercept significantly different from zero (<0.05) were considered unreliable and potentially biased owing to pleiotropy.^[37]^ We also performed a leave-one-out analysis to evaluate the stability of the genetic instruments by sequentially removing each individual SNP and assessing its impact on the overall results.^[38]^ Additionally, the MR-Pleiotropy Residual Sum and Outlier (MR-PRESSO) method was employed to detect and correct horizontal pleiotropy.^[37,39]^ If outliers were identified, they were excluded and the MR causal estimates were reassessed.

Finally, the results were visualized using scatter plots and funnel plots to provide a clear depiction of the MR findings and further assess the presence of pleiotropy or heterogeneity.

### 2.7. Replication MR and meta-analysis

To ensure the robustness of our findings, we conducted a replication MR analysis using sepsis GWAS data from the FinnGen Consortium. Through a meta-analysis of the 2 MR results, we ultimately identified immune cells with a potential impact on sepsis. Fixed-effect and random-effect models were used to combine the MR results derived from the UK Biobank and FinnGen databases.

### 2.8. Bidirectional causality analysis

To evaluate the bidirectional causation effects between immune cells or inflammatory proteins and sepsis, we used sepsis as “exposure” and immune cells or inflammation proteins as “outcome” (Steps 1 and 2 in Fig. 1). We selected SNPs that were significantly associated with sepsis (*P* < 5 × 10^−6^) as IVs.

### 2.9. Genetic correlation analysis

We estimated the genetic correlation (Rg) between immune cells and sepsis by using LDSC. GWAS summary statistics were filtered according to the HapMap3 ref. Variants that were not SNPs (e.g., indels) and SNPs that were strand-ambiguous, repeated, and had a minor allele frequency < 0.01 were excluded. The LDSC examines the association between test statistics and LD to quantify the contribution of inflation from a true polygenic signal or bias.^[40]^ This method can evaluate genetic correlations from GWAS summary statistics and is not biased by sample overlap.^[41]^ The z-scores of each variant from trait 1 were multiplied by the z-scores of each variant from trait 2. Genetic covariance was estimated by regressing the product against the LD score.^[42]^ The genetic covariance normalized by SNP-heritability represents the genetic correlation.

### 2.10. Mediation analysis

Using a 2-sample analysis (Steps 1 and 2 in Fig. 1), the immune cells and inflammatory proteins with significant causal effects on sepsis were included in the mediation analysis to explore whether specific inflammatory proteins served as the mediation factors in the pathway from immune cells to sepsis. To calculate the mediation proportion for each immune cell trait correlated with both inflammatory proteins and sepsis, we multiplied the mediation proportions for β1 and β2 and then divided by the total effect of inflammatory proteins on sepsis. We employed the delta method to derive the 95% confidence interval (CI) for the mediation proportion effect^[43,44]^ (Step 3 in Fig. 1).

All statistical analyses were conducted using the TwoSampleMR (0.6.1), MR-PRESSO (1.0), RMediation (1.2.2), and meta (7.0-0) packages in R, version 4.3.2 (https://www.r-project.org/).

## 3. Results

### 3.1. Impact of immune cells on sepsis

The IVW results identified 41 immune cells associated with sepsis in the discovery cohort (Table S3, Supplemental Digital Content, https://links.lww.com/MD/P612). Techniques including MR-Egger and Weighted median aligned in the direction with the IVW method, reinforcing the robustness of our findings. Scatter plots demonstrate the specific effects of each method on the outcome database. Cochran *Q* test revealed no significant heterogeneity (*P* > .05). Additionally, no significant horizontal pleiotropy was found in the results of the MR-Egger intercept (Table S3, Supplemental Digital Content, https://links.lww.com/MD/P612) and MR-PRESSO analyses (Table S4, Supplemental Digital Content, https://links.lww.com/MD/P612). The leave-one-out analysis further confirmed that causal estimates of immune cells were given. In the discovery cohort, 41 immune cells were found to be associated with sepsis, among which 20 immune cells showed consistent effects in the replication cohort (Table S5, Supplemental Digital Content, https://links.lww.com/MD/P612).

**Figure 2.**
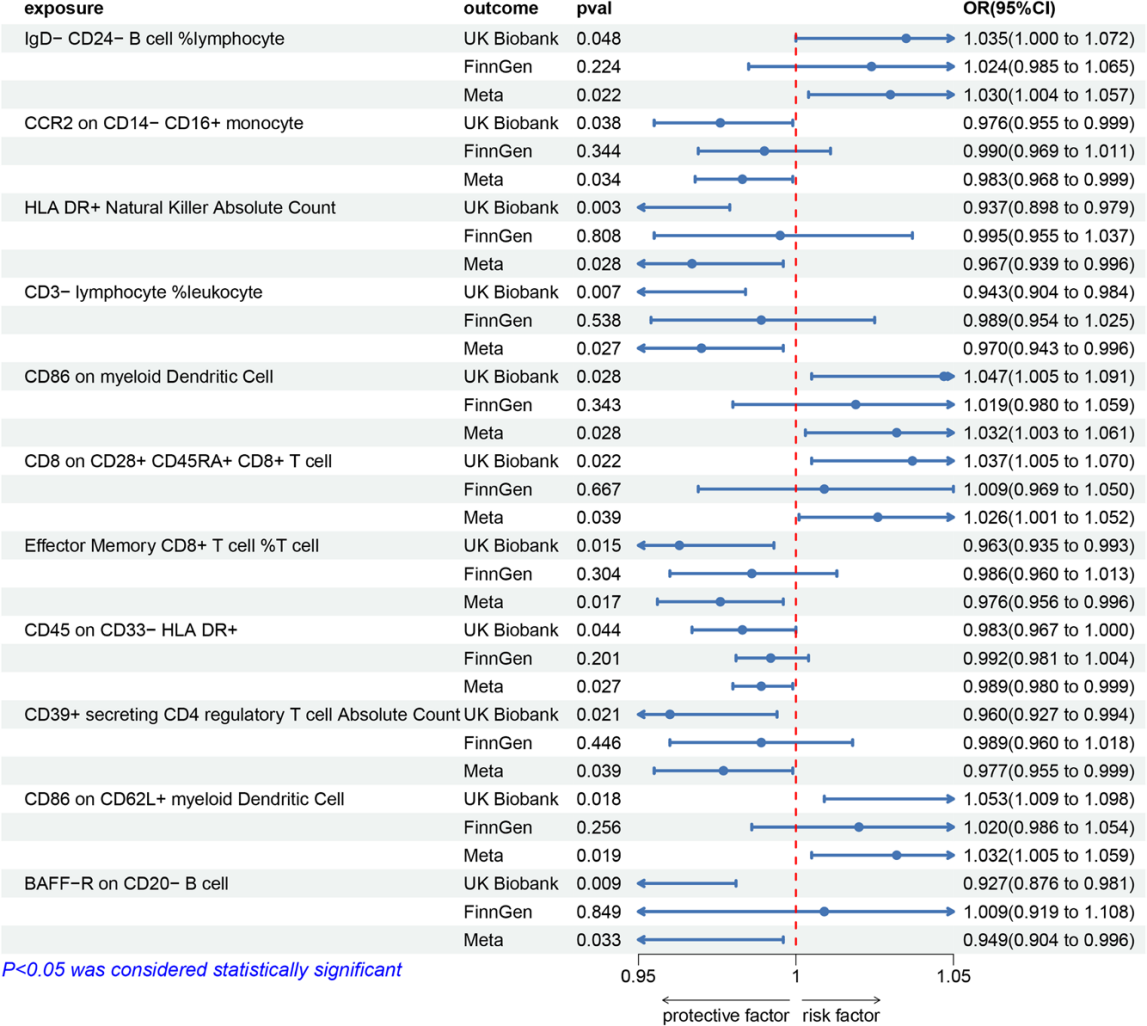
Forest plot to visualize the causal effect of immune cells on sepsis using the inverse variance weighted method and meta-analysis. 95% CI = 95% confidence interval, OR = odds ratio.

Furthermore, meta-analysis results based on the IVW method confirmed that 11 immune cells were associated with sepsis (*P* < .05) (Fig. 2). No inverse causal relationships were found between these 11 immune cell types and sepsis (Table S6, Supplemental Digital Content, https://links.lww.com/MD/P612).

### 3.2. LDSC regression analysis

We performed LDSC regression analysis to evaluate the genetic correlation between the 11 immune cell types and sepsis. Owing to limitations, such as low heritability, some immune cells could not be used for the above analysis. Finally, we identified a small genetic correlation between sepsis and CCR2 in CD14-CD16 + monocytes (Rg = −0.3894, *P* = .4369), CD86 on myeloid dendritic cells (DC) (Rg = −0.9016, *P* = .0825), CD45 on CD33- HLA-DR + (Rg = −6.9193, *P* = .0928), and CD86 on CD62L + myeloid DC (Rg = −0.3989, *P* = .3226) (Table S7, Supplemental Digital Content, https://links.lww.com/MD/P612).

### 3.3. Impact of inflammatory proteins on sepsis

**Figure 3.**
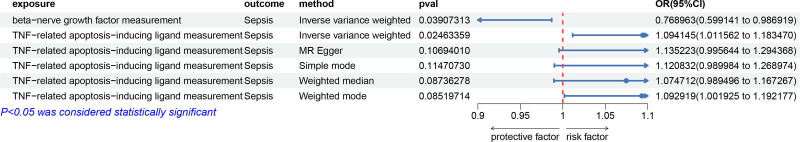
Forest plot to visualize the causal effect of inflammatory proteins on sepsis. 95% CI = 95% confidence interval, OR = odds ratio.

Among the 92 inflammatory proteins, a causal relationship was found between the 2 inflammatory proteins and sepsis. Among these, the measurement of beta-nerve growth factor (OR = 0.769, 95% CI: 0.60–1.01, *P* = .039) was negatively correlated with sepsis, and there was no evidence of heterogeneity among the genetic instruments (*Q* = 0.0723, *P* = .788). In addition, sensitivity analysis could not be performed because of the limited number of SNPs in beta-nerve growth factor. Measurements of TNF-related apoptosis-inducing ligand (TRAIL) (OR = 1.094, 95% CI: 1.01–1.18, *P* = .025) were positively correlated with sepsis. No heterogeneity was detected among the genetic instruments for the measurement of TNF-related apoptosis-inducing ligand (TRAIL: *Q* = 9.947, *P* = .192), and no level of polygenic pleiotropy was observed (TRAIL: Egger intercept = −0.0096, *P* = .51) (Fig. 3 and Table S8, Supplemental Digital Content, https://links.lww.com/MD/P612). No evidence was found to suggest a reverse causal relationship between the 2 aforementioned inflammatory proteins and sepsis (Table S9, Supplemental Digital Content, https://links.lww.com/MD/P612).

### 3.4. Mediation analysis

In this study, we identified 11 immune cells and 2 inflammatory proteins that were associated with sepsis. Initially, it appeared that inflammatory proteins exerted a mediating effect on the pathway between the immune cells and sepsis. However, our results revealed that inflammatory proteins did not act as mediators in the pathways of immune cells and sepsis.

### 3.5. The causal relationship between immune cells and subtypes of sepsis

**Figure 4.**
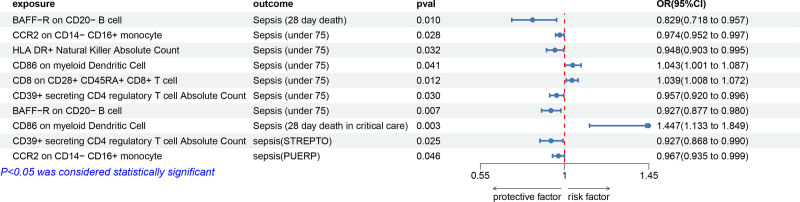
Forest plot to visualize the causal effect of immune cells on subtypes of sepsis using the inverse variance weighted method and meta-analysis. 95% CI = 95% confidence interval, OR = odds ratio.

After conducting a meta-analysis of data from the UK Biobank (UKBB) and FinnGen databases, we identified 11 immune cell types with a causal relationship to sepsis. Notably, 10 of these immune cell types exhibited causal associations with various sepsis subtypes, including Sepsis (28 day death), sepsis (under 75 years), Sepsis (28 day death in critical care), sepsis (critical care), streptococcal septicemia, and puerperal sepsis. Our study revealed that immune cells are protective factors for sepsis subtypes, whereas 3 immune cells are risk factors. Detailed results are presented in Figure 4 and Table S10 (Supplemental Digital Content, https://links.lww.com/MD/P612).

## 4. Discussion

To our knowledge, this is the first study to highlight the role of alterations in specific immune cells in the development of sepsis and to provide insights into the potential mediating effects of inflammatory proteins. Our findings suggest that genetically determined increases in certain immune cell phenotypes, such as CCR2 on CD14 − CD16 + monocytes, HLA-DR + natural killer absolute count, and CD3 − lymphocyte %leukocyte, are associated with a reduced risk of sepsis. Conversely, higher levels of IgD − CD24 − B cell % lymphocytes and CD86 on myeloid DC are associated with an increased risk of sepsis. Additionally, we identified beta-nerve growth factor as a protective factor, and TRAIL as a risk factor for sepsis. However, our analysis did not find evidence that these inflammatory proteins mediate the pathway between the immune cells and sepsis.

Sepsis is a heterogeneous clinical syndrome characterized by a robust systemic inflammatory response that is typically triggered by infection.^[45–48]^ Immune dysfunction involving various types of immune cells is a critical feature of sepsis. The innate immune response acts as the body’s first line of defense against invasive pathogens.^[45]^ Neutrophils and monocytes are among the first responders to recognize pathogen-associated molecular patterns (PAMPs) and damage-associated molecular patterns (DAMPs). These interactions prompt mononuclear phagocytes (MNPs) to release a cascade of cytokines, including TNF-α, interleukin (IL)-1, and IL-6, which serve to recruit and activate additional immune cells.^[45,48]^ While neutrophils are primarily tasked with the direct elimination of microbes, mononuclear phagocytes not only destroy pathogens, but also play a crucial role in presenting their antigenic content to the adaptive immune system, thereby bridging innate and adaptive immune responses.^[48,49]^

Our study identified CCR2 on CD14 − CD16 + monocytes, as participants in the innate immune response, to be inversely associated with the risk of developing sepsis. CCR2 on CD14- CD16 + monocyte links innate and adaptive immune systems by their ability to adopt either pro- or anti-inflammatory functions.^[48,50]^ The diminished expression of human leukocyte antigen-DR (HLA-DR) in blood monocytes is a well-established marker of immunosuppression in sepsis and correlates with poor outcomes.^[51]^ Patients with delayed or no improvement in HLA-DR or a decline in HLA-DR expression have a higher risk of 28-day mortality.^[52]^ decrease in HLA-DR leads to diminished antigen presentation and reduced adaptive immune activation, resulting in immunosuppression and poor outcomes.^[48,53,54]^ Multiple studies support mHLA-DR expression by flow cytometry as a sepsis immunoparalysis biomarker and mortality predictor.^[55]^ Our study corroborates the findings of previous observational studies by demonstrating that diminished expression of CD45 on CD33 − HLA-DR + cells may be associated with an increased risk of sepsis onset. Our research findings indicate that elevated levels of HLA-DR in the HLA-DR + NK circulating immune cell phenotype are associated with a decreased risk of sepsis. The HLA-DR + NK immunophenotype represents a specific subtype of NK cells expressing the HLA-DR molecule. NK cells play an immunoregulatory role by secreting cytokines when they are stimulated^[56]^; therefore, both impaired NK cell function and a reduction in NK cell counts may be part of an immunodeficiency that makes injured patients susceptible to sepsis. It has been shown that the frequency of HLA-DR + NK cells in survivors showed an upward-regulated trend, while there was no significant change in non-survivors, suggesting a continuous compensatory increase in activated NK cells in survivors.^[57]^

“CD3- lymphocyte %leukocyte” refers to the proportion of lymphocytes within the total leukocyte population that do not express the CD3 molecule. Previous observational studies have reported that a diminished percentage of circulating γδT cells may contribute to an increased incidence of secondary infections.^[58]^ Our study further supports the findings of Matsushima et al,^[59]^ who reported a reduced percentage of circulating γδT cells in patients with sepsis, which may be linked to compromised immune function. B cell-activating factor (BAFF) is a crucial survival and maturation factor for B cells and belongs to the tumor necrosis factor superfamily. Among the 3 identified functional receptors, the BAFF receptor (BAFF-R) is primarily responsible for B cell survival and differentiation.^[60]^ Studies have demonstrated that BAFF-mediated stimulation can inhibit apoptosis induced by signals through CD20 and B-cell receptors (BCR), thereby enhancing B cell survival.^[61]^ Our study is consistent with the findings of Saito et al, suggesting that BAFF-R on CD20 − B cells may play a protective role against sepsis by mediating B cell survival and bolstering the immune system’s resistance to foreign pathogens.

“Effector Memory CD8 + T cell %T cell” refers to the proportion of CD8 + T-cells within the total T cell population that exhibits effector memory characteristics. These cells are crucial in the immune response because of their ability to rapidly and robustly defend against previously encountered pathogens. Patients with sepsis have an enhanced susceptibility to both acute and chronic infections. This increased vulnerability is partly due to impaired T-cell responses in septic hosts, leading to a reduced number of effector CD8 + T cells capable of producing key effector cytokines, such as IFN-γ, TNF-α, and IL-2.^[62,63]^ Our study also confirms that a higher proportion of Effector Memory CD8 + T cells serves as a protective factor against sepsis. “CD39 + secreting CD4 regulatory T cell Absolute Count” refers to the absolute quantification of CD4 + regulatory T cells (Treg cells) in the bloodstream that express CD39 and possess secretory capabilities. These Tregs are instrumental in modulating immune responses and promoting immune tolerance. Upon recognizing cognate antigens presented by antigen-presenting cells (APCs), naive CD4 + T cells differentiate into various phenotypes, including T-helper 1 (Th1), Th2, Th17, and regulatory T cells (Tregs), which are influenced by environmental cytokines.^[64–70]^ Tregs play a vital role in maintaining immune homeostasis, limiting excessive immune responses after infection to prevent tissue damage.^[71]^ The presence of CD39+-secreting CD4 regulatory T cells may act as a protective factor for patients with sepsis by preventing excessive immune responses that could lead to self-inflicted harm.

IgD− CD24− B cell % lymphocytes refer to the percentage of B cells within the lymphocyte population that do not express IgD or CD24 on their surface. CD24, when engaged with Siglec-g/10 on macrophage surfaces, interacts with DAMPs through immunoreceptor tyrosine-based inhibitory motif (ITIM) signaling, thereby inhibiting inflammatory processes. Disruption of this anti-inflammatory “braking” mechanism, such as through the presence of CD24 antibodies, can lead to a cytokine storm.^[72–78]^ The absence of CD24 and IgD expression on B cells may contribute to immune evasion and the occurrence of cytokine storms in patients with sepsis, potentially explaining our finding that IgD − CD24 − B cells are associated with an increased risk of sepsis. “CD8 on CD28 + CD45RA + CD8 + T cells “ refers to CD8 + T cells that co-express CD8, CD28, and CD45RA on their surfaces. Following pathogen invasion, the expression of both CD28 + and CD45RA + was significantly upregulated, suggesting that co-expression of these markers on CD8 + T cells may play a role in the inflammatory response.^[79,80]^ Based on this, our study hypothesized that CD8 + CD28 + CD45RA + CD8 + T cells may contribute to the pathogenesis and progression of sepsis through these mechanisms, suggesting that this subset of T cells could be a risk factor for sepsis.

Both CD86 on myeloid DC and CD86 on CD62L + myeloid DC are subsets of myeloid dendritic cell lineage. Dendritic cells (DC) are a heterogeneous population of professional antigen-presenting cells that play a pivotal role in the initiation and modulation of innate and adaptive immune responses.^[81,82]^ Several studies in murine models strongly suggest that deviated DC behavior could account for the immunosuppression observed during sepsis.^[83–86]^ Our study indicated that CD86 + myeloid DC (mDCs) and CD62L + mDCs are risk factors for sepsis, suggesting that these cell populations may disrupt immune regulation, potentially leading to immunosuppression and secondary infections, which could precipitate sepsis and ultimately impact patient recovery and prognosis.

The study elucidates the critical role of protective immune cell phenotypes, such as CCR2 + CD14 − CD16 + monocytes and HLA-DR + NK cells, in modulating sepsis progression through coordinated innate and adaptive immune responses. CCR2^+^ monocytes migrate to infection sites via CCR2 and differentiate into anti-inflammatory M2 macrophages, thereby suppressing cytokine storms.^[87]^ In contrast, HLA-DR^+^ NK cells enhance pathogen clearance by promoting macrophage activation via elevated IFN-γ secretion.^[88]^ Paradoxically, risk factors such as heightened CD86^+^ myeloid dendritic cell (mDC) expression may excessively activate T cells, triggering aberrant release of pro-inflammatory cytokines (IL-6, TNF-α) and subsequent tissue damage.^[89]^ β-Nerve growth factor (β-NGF), which exhibits dual neurotrophic and immunomodulatory functions, mitigates sepsis-associated hyperinflammation by suppressing the NF-κB pathway.^[90]^ Although inflammatory proteins (e.g., TRAIL) constitute independent risk factors, they do not mediate immune cell-sepsis associations. This implies that immune cells likely regulate sepsis pathogenesis through non-canonical pathways – such as mitochondrial DNA release or pyroptosis – or direct intercellular interactions.^[91]^ Given the complexity of sepsis immunopathology, immune cell subsets demonstrate significant functional heterogeneity during disease initiation and progression.^[92]^ Further investigation into non-classical inflammatory mechanisms is thus essential to comprehensively decipher their roles. Future investigations should focus on in vitro co-culture models, genetically engineered animal models, single-cell transcriptomic validation, and targeted intervention studies to further elucidate the functional roles of these immune phenotypes and their therapeutic potential in sepsis.

Our study has several strengths, including the large sample size, use of multiple MR methods to ensure robustness, and leveraging of GWAS data to minimize confounding factors. Sensitivity analyses using different statistical models yielded consistent results, further supporting the reliability of the conclusions. However, our study had some limitations. First, the threshold for selecting IVs was relatively lenient (1 × 10 − 5), which may introduce false positives and biases. Second, our findings are based on data from individuals of European descent, and further research is required to determine their generalizability to other ethnic groups. In addition, translating these genetic correlations into practical clinicaltreatments requires further in-depth exploration. Finally, although we investigated the mediating effects of inflammatory proteins between immune cells and sepsis, the mechanisms by which immune cells influence the onset of sepsis remain to be elucidated, given that inflammatory proteins did not appear to act as mediating factors in our study.

## 5. Conclusion

This study provides genetic support for the relationship among immune cells, inflammatory proteins, and sepsis. However, inflammatory proteins did not appear to mediate the pathway between the immune cells and sepsis. Further experimental research is warranted to validate the observed associations and to elucidate the underlying biological mechanisms linking immune cells to sepsis development. Clinical studies with diverse patient populations are needed to confirm the prognostic utility of these immune cells. Overall, our findings underscore the pivotal role of immune cells in the pathogenesis of sepsis and justify further exploration of potential immunotherapeutic targets.

## Acknowledgments

We would like to thank the researchers and study participants for their contributions.

## Author contributions

**Conceptualization:** An Zhang.

**Data curation:** Yan Zhang, Weiwei Xu, An Zhang.

**Formal analysis:** An Zhang.

**Funding acquisition:** An Zhang.

**Methodology:** Wenqi Huang.

**Project administration:** Wenqi Huang.

**Resources:** Wenqi Huang.

**Software:** Yan Zhang.

**Supervision:** Wenqi Huang, An Zhang.

**Validation:** Yan Zhang, Weiwei Xu.

**Visualization:** Yan Zhang.

**Writing – original draft:** Yan Zhang.

**Writing – review & editing:** Yan Zhang, Weiwei Xu.

## Supplementary Material


